# Phase 1 study of talquetamab, a humanized GPRC5D x CD3 bispecific antibody, in Japanese patients with relapsed/refractory MM

**DOI:** 10.1007/s12185-025-03991-5

**Published:** 2025-05-09

**Authors:** Shinsuke Iida, Kazutaka Sunami, Shigeki Ito, Junichiro Yuda, Ei Fujikawa, Mikihiro Takamoto, Kensuke Aida, Hiroshi Yamazaki, Marimo Takahashi, Tadao Ishida

**Affiliations:** 1 Department of Hematology and Oncology, Nagoya City University Graduate School of Medical Sciences, Nagoya, Japan; 2 Department of Hematology, NHO Okayama Medical Center, Okayama, Japan; 3 Hematology and Oncology, Department of Internal Medicine, Iwate Medical University School of Medicine, Yahaba-Cho, Japan; 4 Department of Hematology, National Cancer Center Hospital East, Kashiwa, Japan; 5 Janssen Pharm KK, R&D, Tokyo, Japan; 6 Department of Hematology, Japanese Red Cross Medical Center, Tokyo, Japan

**Keywords:** Immunotherapy, Japanese population, Relapsed/refractory multiple myeloma, Talquetamab

## Abstract

The bispecific antibody talquetamab demonstrated substantial responses in heavily pretreated relapsed or refractory multiple myeloma (RRMM) in the global phase 1/2 MonumenTAL-1 study. This study, evaluated the safety and efficacy of talquetamab in Japanese patients with RRMM pretreated with a proteasome inhibitor, immunomodulatory drug, and anti-CD38 monoclonal antibody. The primary endpoints were frequency and type of treatment-emergent adverse events (TEAEs) and serious AEs including dose-limiting toxicity (DLT). The secondary endpoints were overall response (ORR; partial response or better), duration of, and time to response. At data cutoff, 15 patients had received subcutaneous talquetamab at three doses (Cohort 1: 135 µg/kg weekly [QW, n = 4]; Cohort 2: 400 µg/kg [QW, n = 5]; Cohort 3: 800 µg/kg [Q2W, n = 6]). No DLTs, deaths, or AE-related dose reductions/treatment discontinuation were observed. Common TEAEs were neutropenia (60.0%), lymphopenia (53.3%), and CRS (46.7%). TEAEs of clinical interest (all Grade ≤ 2) were dysgeusia, skin toxicity, nail disorder, and dry mouth. With an overall median follow-up of 9.0 months, the ORR was 60.0% (95% confidence interval 32.3%, 83.7%). Talquetamab showed substantial responses in Japanese patients with RRMM, consistent with the global MonumenTAL-1 study, supporting its potential as a new standard of care for Japanese RRMM patients.

**Supplementary Information:**

The online version contains supplementary material available at 10.1007/s12185-025-03991-5.

## Introduction

Multiple Myeloma (MM) is a genetically complex and biologically heterogenous disease where nearly all patients relapse even after initial standard treatment [1, 2]. In Japan, every year MM accounts for about 4000 deaths [3] with a slight rise in the age-adjusted incidence rate from 2016 to 2020 (5.96.1 per 100,000 people) [4, 5]. Since the early 2000 s, several novel treatment modalities have been approved in Japan for the management of MM, leading to improved overall survival (OS) [6]. Despite these advancements, relapse remains a significant challenge, particularly for triple-class exposed (who have been treated and are refractory to a combination of immunomodulatory drugs [IMiDs], proteasome inhibitor [PIs], and anti-CD38 antibodies) relapsed/refractory MM (RRMM) [7, 8]. The patients with triple-class exposed RRMM face treatment challenges with only 29.8% having a response with a median OS of 12.4 months [9]. While recent therapies for triple-class exposed RRMM have resulted in improved outcomes [10–15], myeloma patients will eventually relapse. Therefore, additional novel therapies with distinct mechanisms of action are required.

Innovative therapies such as T cell-redirecting bispecific antibodies (BsAb) have demonstrated high response rates, showcasing their potential in modern treatment approaches for MM. Among these, talquetamab, is a first in class BsAb that binds to CD3 on T cells and redirects T cells to kill malignant plasma cells expressing G protein-coupled receptor class C Group 5 member D (GPRC5D). Based on the encouraging outcomes from the global MonumenTAL-1 study, talquetamab has received approval for treatment of RRMM in both United States and Europe [16–18]. The MonumenTAL-1 study focused primarily on the efficacy and safety of talquetamab for treating RRMM patients with multiple prior treatments including a PI, an IMiD, and an anti-CD38 antibody. The study identified 2 recommended phase 2 doses (RP2D) delivered subcutaneously (SC), 400 µg/kg administered every week (QW), and 800 µg/kg administered every 2 weeks (Q2 W). The results of this pivotal phase-1/2 study showed that at data-cutoff of January 17, 2023, at median follow-ups of 18.8 months, (for patients receiving talquetamab at the 400-μg/kg QW) and 12.7 months (for patients receiving it at the 800-μg/kg Q2 W), the response rates were 74.1% and 71.7%, respectively. The median duration of response (DOR) was 9.5 months (95% CI 6.7, 13.3) at 400-μg/kg QW and not reached (NR) (95% CI 13.0, NE) at 800-μg/kg Q2 W. The common adverse events (AEs) such as cytokine release syndrome (CRS), skin toxicity, and dysgeusia were effectively managed, highlighting its potential as a viable treatment option [19].

Here we report the safety, tolerability, and preliminary efficacy results of phase 1 talquetamab study in heavily pretreated Japanese patients with triple-class exposed RRMM.

## Methods

### Study design and treatment

This phase-1, open-label, multicenter dose escalation, study was designed to evaluate the safety, tolerability, and preliminary efficacy of talquetamab including RP2D in Japanese patients with RRMM. The study consisted of 2 periods, a screening period that began 28 days prior to talquetamab administration and a treatment period, which started with the initial dose of the study treatment and lasted until the end-of-treatment visit (defined as within 30 days [+ 7 days] after the last dose of study treatment or prior to the start of a new anticancer therapy, whichever comes first) was completed.

The patients were divided into 3 Cohorts to receive talquetamab at different doses (Cohort 1:135 µg/kg QW], Cohort 2: 400 µg/kg QW, Cohort 3: 800 µg/kg Q2 W). Dose escalation started with Cohort 1, followed by Cohort 2, and Cohort 3. Notably, Cohort 3 was introduced after study initiation, as 800 µg/kg at Q2 W was newly identified as RP2D in global MonumenTAL-1 study. Cohort 1 and Cohort 2 received SC treatment doses on days 1, 8, and 15 of a 21-day Cycle. The step-up schedule started about 7 days before the first treatment dose, with 2 doses [135 µg/kg: 10 µg and 45 µg; 400 µg/kg: 10 µg and 60 µg)] given 2–4 days apart. Cohort 3 received SC talquetamab on days 1 and 15 of a 28-day Cycle and the step-up schedule started about 7 days before the first treatment dose, with 3 doses (10 µg, 60 µg, and 300 µg) given 2–4 days apart. Patients were required to receive glucocorticoid, antihistamine, and antipyretic medications prior and on the day of study treatment administration at least during step-up doses and at Cycle 1 day 1, in order to mitigate CRS. Talquetamab dose modifications were permitted for patients who experienced talquetamab-related toxicities and met the criteria specified in study protocol. Patients continued to receive treatment until 1 of the study treatment discontinuation conditions was met i.e., disease progression, unacceptable toxicity, withdrawal of consent, or end of study treatment.

### Patients

The key eligibility criteria included adult patients (aged ≥ 20 years) with a documented diagnosis of MM according to International Myeloma Working Group (IMWG) diagnostic criteria [20]; measurable disease (M-protein level, Serum: ≥ 1.0 g/dL/Urine: ≥ 200 mg/24 h) or light chain multiple myeloma (≥ 100 mg/L free light chain (FLC) with abnormal FLC ratio for patients without measurable disease in the serum or urine): Serum immunoglobulin-free; Eastern Cooperative Oncology Group (ECOG) performance status of 0 or 1; relapsed or refractory to established therapies, and a platelet count of at least 50,000 per cubic millimeter, an absolute neutrophil count of ≥ 1000 cells per cubic millimeter and a creatinine clearance of at least 40 mL per minute per 1.73 m^2^ of body-surface area. Patients were required to have received a previous PI, an IMiD, and an anti-CD38 antibody in any order during the course of treatment. The study allowed patients who could not tolerate a PI, IMiD or anti-CD38 antibody. Patients previously affected by Grade 3 CRS from T cell therapies (e.g., CAR-T or CD-3 redirection technology) or treated with GPRC5D-targeted therapies were excluded. During the study, supportive care measures like antibiotic prophylaxis (antivirals, antibacterials and vaccines), immunoglobulin replacement therapy, and granulocyte colony- stimulating factor were provided to the patients.

### Endpoints and assessments

The primary endpoint of the study was to assess frequency and type of AEs and serious adverse events (SAEs) including dose-limiting toxicity (DLT). DLT was evaluated from the 1 st step-up dose to the end of the first Cycle (Cohort 1 and 2: ≥ 28 days; Cohort 3: ≥ 38 days), concluding immediately before the first dose of Cycle 2. The criteria for DLTs are outlined in Supplementary Table S1.

Secondary endpoints were to evaluate pharmacokinetics (PK) parameters and pharmacodynamic (PD) markers including cytokine concentrations, incidence of anti-talquetamab antibodies and preliminary antitumor activity including overall response (partial response [PR] or better), DOR and time to response (TTR) assessed by investigator and as defined by the IMWG response criteria in MM, of talquetamab.

The severity of AEs was assessed using National Cancer Institute Common Terminology Criteria for AEs (NCI-CTCAE, Version 5.0), with the exception of CRS and Immune effector cell-associated neurotoxicity (ICANS), which were evaluated according to American Society for Transplantation and Cellular Therapy (ASTCT) guidelines [21], AEs were coded using the MedDRA terminology (Version 26.1).

### Statistical analysis

Dose escalation was guided by Bayesian optimal interval (BOIN) design. Safety and efficacy were assessed in patients who had received at least one dose of talquetamab. In the response analysis, the percentage of patients with a PR or better was calculated with a two-sided 95% exact confidence interval (CI). Patients with no post-baseline response data will be considered as non-responders. Data were summarized with the use of descriptive statistics and Kaplan–Meier methods were used to estimate DOR. The data-cutoff date was February 09, 2024, for the safety and efficacy analyses. All data analyses were performed, and outputs were generated using SAS version 9.4.

### Pharmacokinetic evaluations

Serum concentrations of talquetamab were analyzed using a validated, specific, and sensitive immunoassay method. The lower limit of quantification (LLOQ) for the serum concentrations of talquetamab were 0.500 ng/mL.

## Results

### Patient disposition

**Fig. 1:**
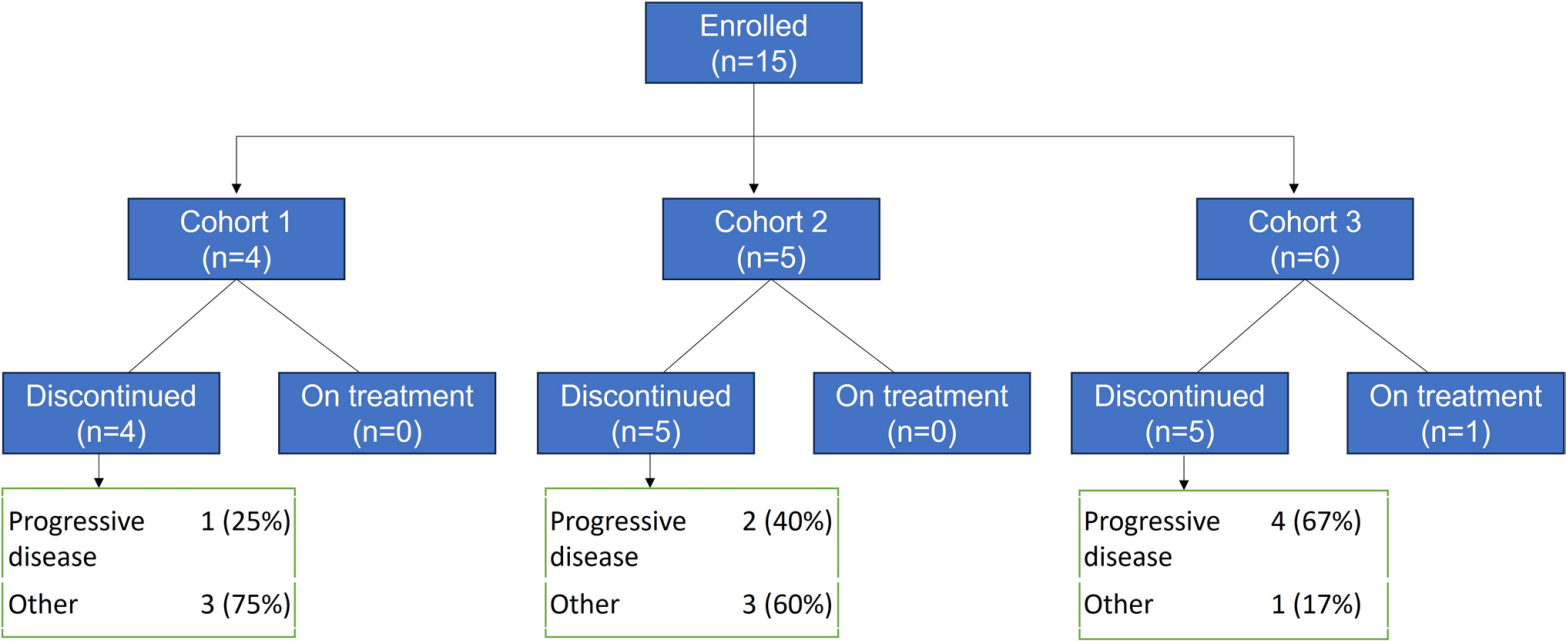
Patient disposition. All patients in Cohort 1 and 2 patients in Cohort 2: After disease progression as per the IMWG criteria, the patients received ethical supply, but the administration was discontinued due to further disease progression. One patient each in Cohort 2 and 3: Withdrawal by patient

Overall, 19 patients were screened for the study inclusion across 6 sites in Japan from May 2021. At the clinical data-cutoff date of February 09, 2024, a total of 15 patients received ≥ 1 dose of SC talquetamab. These patients were grouped into 3 Cohorts (Cohort 1 [n = 4]; Cohort 2 [n = 5]; Cohort 3 [n = 6]). A total of 14 patients discontinued the study treatment due to disease progression (n = 7, 46.7%), or due to other reasons (n = 7, 46.7%) (Fig. 1). A total of 5 patients out of 7 who discontinued due to other reasons continued talquetamab after confirmed progressive disease for ethical reason but discontinued due to further aggregation of primary disease, while 2 patients discontinued after withdrawal of the consent (Fig. 1).

### Baseline characteristics

The median age of the patients was 65 years (range, 45–86) with 40% (n = 6) of these were ≥ 70 years old. A total of 66.7% (n = 10/15) of patients were at high-risk cytogenetic profile [del(17p), t(4:14), t(14:16)]. Patients received a median of 5.0 (range, 2–16) prior lines of therapy; 73.3% (11/15) of them received prior penta-drug therapy. A total of 80.0% (12/15) patients were triple-class refractory; 40.0% (6/15) were penta-drug refractory, while 73.3% (11/15) were refractory to last line of prior therapy. A total of 20% (3/15) patients had previously undergone anti BCMA-targeted chimeric antigen receptor-T cell (BCMA CAR-T cell) therapy and 6.7% (1/15) with BsAb therapy (Table 1; Supplementary Table S7).

**Table 1:** Summary of demographic and baseline characteristics

Characteristics	Cohort 1 (n = 4)	Cohort 2 (n = 5)	Cohort 3 (n = 6)	Total (N = 15)
Age (years); median (range)	60.0 (45–77)	60.0 (56–81)	69.5 (61–86)	65.0 (45–86)
≥ 70 years, n (%)	1 (25.0)	2 (40.0)	3 (50.0)	6 (40.0)
Sex (female), n (%)	1 (25.0)	2 (40.0)	4 (66.7)	7 (46.7)
Median time from MM diagnosis to first step-up dose (range), years	5.92 (1.5, 7.1)	3.57 (2.8, 14.1)	5.69 (1.4, 23.9)	5.65 (1.4, 23.9)
≥ 60% plasma cells in bone marrow aspirate, n (%)	2 (50.0)	0	0	2 (13.3)
International Staging System class, n (%)^*^				
I	1 (25.0)	4 (80.0)	2 (33.3)	7 (46.7)
II	2 (50.0)	1 (20.0)	4 (66.7)	7 (46.7)
III	1 (25.0)	0	0	1 (6.7)
High-risk cytogenetic abnormalities, n (%)^†^				
del(17p)	1 (25.0)	2 (40.0)	3 (50.0)	6 (40.0)
t(4;14)	0	3 (60.0)	1 (16.7)	4 (26.7)
t(14;16)	1 (25.0)	0	0	1 (6.7)
Median no. of lines of previous therapy (range)	6.5 (4, 8)	5.0 (3, 8)	5.0 (2, 16)	5.0 (2, 16)
Previous stem-cell transplantation, n (%)	3 (75.0)	3 (60.0)	3 (50.0)	9 (60.0)
Previous BCMA-targeted/BsAb therapy, n (%)				
CAR-T cell	2 (50.0)	1 (20.0)	0	3 (20.0)
BsAb	1 (25.0)	0	0	1 (6.7)
Previous therapy exposure, n (%)				
Triple-class exposure^§^	4 (100.0)	5 (100.0)	6 (100.0)	15 (100.0)
Penta-drug exposure^¶^	3 (75.0)	3 (60.0)	5 (83.3)	11 (73.3)
Refractory status, n (%)				
Immunomodulatory drug^‖^	4 (100.0)	5 (100.0)	6 (100.0)	15 (100.0)
Proteasome inhibitor^**^	3 (75.0)	4 (80.0)	6 (100.0)	13 (86.7)
Anti-CD38 monoclonal antibody^††^	4 (100.0)	4 (80.0)	6 (100.0)	14 (93.3)
Triple-class refractory^‡‡^	3 (75.0)	3 (60.0)	6 (100.0)	12 (80.0)
Penta-drug refractory^§§^	1 (25.0)	1 (20.0)	4 (66.7)	6 (40.0)
Refractory to last line of therapy	3 (75.0)	2 (40.0)	6 (100.0)	11 (73.3)

Treatment schedule: Cohort 1: 135 μg/kg SC QW, Cohort 2: 400 μg/kg SC QW, and Cohort 3: 800 μg/kg SC Q2 W. There were no ≥ 1 Extramedullary plasmacytoma

^*^ISS staging is derived based on serum β2-microglobulin and albumin

^†^Patients had high-risk cytogenetic abnormalities if they had del(17p), t(4;14), t(14;16), or all these profiles on the basis of local testing

^§^Triple-class exposure was defined as previous treatment with at least one proteasome inhibitor, at least one immunomodulatory drug, and at least one anti-CD38 antibody

^¶^Penta-drug exposure was defined as previous treatment with at least 2 proteasome inhibitors, at least 2 immunomodulatory drugs, and at least one anti-CD38 antibody

^‖^Immunomodulatory drugs included thalidomide, lenalidomide, and pomalidomide

^**^Proteasome inhibitors included bortezomib, carfilzomib, and ixazomib

^††^Anti-CD38 monoclonal antibodies included daratumumab and isatuximab

^‡‡^Triple-class refractory was defined as refractory to at least one proteasome inhibitor, at least one immunomodulatory drug, and at least one anti-CD38 antibody

^§§^Penta-drug refractory was defined as refractory to at least 2 proteasome inhibitors, at least 2 immunomodulatory drugs, and at least one anti-CD38 antibody

*BCMA* B-cell maturation antigen, *BsAb* bispecific antibody, *CAR-T* chimeric antigen receptor T cell therapy, *CD-38* Cluster of differentiation 38, *MM* multiple myeloma, *QW* every week, *Q2 W* every 2 weeks

### Safety

No patient reported any DLT. Of the 15 patients included in safety analysis, all patients (100%) had at least 1 TEAE and 14 patients (93.3%) had at least 1 Grade 3 or 4 TEAE post talquetamab dose (Table 2). In Cohorts 2 and 3, all patients (5 in Cohort 2 and 6 in Cohort 3) treated at the RP2D experienced TEAEs, with 5 (100% [Cohort 2]) and 5 patients (83.3% [Cohort 3]) experiencing Grade 3 or 4 TEAEs respectively (Table 2). All patients received supportive therapies. The most frequently used anti-infective prophylaxis included antivirals (acyclovir [100.0%]), antibacterials (sulfamethoxazole + trimethoprim [86.7%]; levofloxacin [33.3%]), and vaccines (COVID-19 [66.7%]). Immunoglobulin replacement therapy was administered to 3 (20.0%) patients, and blood transfusions were given to 4 (26.7%) patients, mainly packed red blood cells (4 [26.7%]) and platelets (1 [6.7%]). Colony-stimulating factors were administered to 8 (53.3%) patients primarily filgrastim (7 [46.7%]) followed by lenograstim (1 [6.7%]). Treatment schedule: Cohort 1: 135 μg/kg SC QW, Cohort 2: 400 μg/kg SC QW, and Cohort 3: 800 μg/kg SC Q2 W. Percentages in the total columns and toxicity grade columns are calculated with the number of patients in each treatment group as denominator

**Table 2:** Treatment-emergent adverse events by system organ class, preferred term and toxicity Grade 3 or 4

	Cohort 1 (n = 4)		Cohort 2 (n = 5)		Cohort 3 (n = 6)		Total (N = 15)	
Events, n (%)	Any grade	Grade 3/4	Any grade	Grade 3/4	Any grade	Grade 3/4	Any grade	Grade 3/4
≥ 1 TEAEs	4 (100.0)	4 (100.0)	5 (100.0)	5 (100.0)	6 (100.0)	5 (83.3)	15 (100.0)	14 (93.3)
Blood and lymphatic system disorders	4 (100.0)	4 (100.0)	5 (100.0)	4 (80.0)	5 (83.3)	5 (83.3)	14 (93.3)	13 (86.7)
Neutropenia	2 (50.0)	2 (50.0)	2 (40.0)	2 (40.0)	5 (83.3)	5 (83.3)	9 (60.0)	9 (60.0)
Lymphopenia	2 (50.0)	2 (50.0)	3 (60.0)	3 (60.0)	3 (50.0)	3 (50.0)	8 (53.3)	8 (53.3)
Anemia	2 (50.0)	2 (50.0)	3 (60.0)	2 (40.0)	1 (16.7)	1 (16.7)	6 (40.0)	5 (33.3)
Leukopenia	1 (25.0)	0	2 (40.0)	1 (20.0)	1 (16.7)	1 (16.7)	4 (26.7)	2 (13.3)
Thrombocytopenia	2 (50.0)	2 (50.0)	0	0	1 (16.7)	1 (16.7)	3 (20.0)	3 (20.0)
General disorders and administration site conditions	1 (25.0)	0	4 (80.0)	0	5 (83.3)	0	10 (66.7)	0
Pyrexia	1 (25.0)	0	2 (40.0)	0	4 (66.7)	0	7 (46.7)	0
Injection site erythema	0	0	2 (40.0)	0	2 (33.3)	0	4 (26.7)	0
Malaise	0	0	2 (40.0)	0	0	0	2 (13.3)	0
Injection site warmth	0	0	0	0	1 (16.7)	0	1 (6.7)	0
Nervous system disorders	3 (75.0)	0	3 (60.0)	0	4 (66.7)	0	10 (66.7)	0
Hypogeusia	2 (50.0)	0	2 (40.0)	0	1 (16.7)	0	5 (33.3)	0
Dysgeusia	1 (25.0)	0	1 (20.0)	0	2 (33.3)	0	4 (26.7)	0
Dyskinesia	0	0	1 (20.0)	0	0	0	1 (6.7)	0
Taste disorder	0	0	0	0	1 (16.7)	0	1 (6.7)	0
Gastrointestinal disorders	1 (25.0)	0	4 (80.0)	0	4 (66.7)	0	9 (60.0)	0
Diarrhoea	0	0	2 (40.0)	0	2 (33.3)	0	4 (26.7)	0
Nausea	0	0	3 (60.0)	0	1 (16.7)	0	4 (26.7)	0
Dysphagia	1 (25.0)	0	1 (20.0)	0	1 (16.7)	0	3 (20.0)	0
Vomiting	0	0	1 (20.0)	0	1 (16.7)	0	2 (13.3)	0
Abdominal pain	0	0	1 (20.0)	0	0	0	1 (6.7)	0
Aphthous ulcer	0	0	0	0	1 (16.7)	0	1 (6.7)	0
Constipation	0	0	1 (20.0)	0	0	0	1 (6.7)	0
Dry mouth	0	0	1 (20.0)	0	0	0	1 (6.7)	0
Gastroesophageal reflux disease	0	0	0	0	1 (16.7)	0	1 (6.7)	0
Irritable bowel syndrome	0	0	0	0	1 (16.7)	0	1 (6.7)	0
Stomatitis	1 (25.0)	0	0	0	0	0	1 (6.7)	0
Skin and subcutaneous tissue disorders	2 (50.0)	0	3 (60.0)	0	4 (66.7)	0	9 (60.0)	0
Alopecia	1 (25.0)	0	0	0	3 (50.0)	0	4 (26.7)	0
Skin exfoliation	1 (25.0)	0	1 (20.0)	0	2 (33.3)	0	4 (26.7)	0
Eczema	1 (25.0)	0	1 (20.0)	0	0	0	2 (13.3)	0
Nail disorder	0	0	1 (20.0)	0	1 (16.7)	0	2 (13.3)	0
Dermatitis bullous	0	0	1 (20.0)	0	0	0	1 (6.7)	0
Dry skin	0	0	0	0	1 (16.7)	0	1 (6.7)	0
Eczema nummular	0	0	0	0	1 (16.7)	0	1 (6.7)	0
Erythema	0	0	1 (20.0)	0	0	0	1 (6.7)	0
Hand dermatitis	1 (25.0)	0	0	0	0	0	1 (6.7)	0
Nail discoloration	0	0	0	0	1 (16.7)	0	1 (6.7)	0
Onychomadesis	0	0	0	0	1 (16.7)	0	1 (6.7)	0
Pruritus	0	0	1 (20.0)	0	0	0	1 (6.7)	0
Rash	0	0	0	0	1 (16.7)	0	1 (6.7)	0
Rash maculo-papular	0	0	0	0	1 (16.7)	0	1 (6.7)	0
Immune system disorders	0	0	2 (40.0)	0	6 (100.0)	0	8 (53.3)	0
Cytokine release syndrome	0	0	2 (40.0)	0	5 (83.3)	0	7 (46.7)	0
Hypogammaglobulinemia	0	0	0	0	1 (16.7)	0	1 (6.7)	0
Infections and infestations	2 (50.0)	1 (25.0)	3 (60.0)	0	3 (50.0)	0	8 (53.3)	1 (6.7)
Upper respiratory tract infection	0	0	1 (20.0)	0	1 (16.7)	0	2 (13.3)	0
COVID-19	0	0	1 (20.0)	0	0	0	1 (6.7)	0
Dermatophytosis of nail	0	0	0	0	1 (16.7)	0	1 (6.7)	0
Gastroenteritis	1 (25.0)	1 (25.0)	0	0	0	0	1 (6.7)	1 (6.7)
Herpes zoster	1 (25.0)	0	0	0	0	0	1 (6.7)	0
Nasopharyngitis	0	0	1 (20.0)	0	0	0	1 (6.7)	0
Otitis media	0	0	1 (20.0)	0	0	0	1 (6.7)	0
Pneumonia	0	0	1 (20.0)	0	0	0	1 (6.7)	0
Tinea pedis	0	0	0	0	1 (16.7)	0	1 (6.7)	0
Metabolism and nutrition disorders	2 (50.0)	1 (25.0)	1 (20.0)	0	2 (33.3)	0	5 (33.3)	1 (6.7)
Hyperglycemia	1 (25.0)	1 (25.0)	1 (20.0)	0	0	0	2 (13.3)	1 (6.7)
Hypozincemia	0	0	1 (20.0)	0	1 (16.7)	0	2 (13.3)	0
Decreased appetite	0	0	0	0	1 (16.7)	0	1 (6.7)	0
Hyperammonemia	1 (25.0)	0	0	0	0	0	1 (6.7)	0
Musculoskeletal and connective tissue disorders	1 (25.0	0	2 (40.0)	0	1 (16.7)	0	4 (26.7)	0
Arthralgia	0	0	1 (20.0)	0	0	0	1 (6.7)	0
Joint range of motion decreased	0	0	1 (20.0)	0	0	0	1 (6.7)	0
Muscular weakness	0	0	0	0	1 (16.7)	0	1 (6.7)	0
Myalgia	1 (25.0)	0	0	0	0	0	1 (6.7)	0
Neck pain	0	0	1 (20.0)	0	0	0	1 (6.7)	0
Spinal deformity	0	0	1 (20.0)	0	0	0	1 (6.7)	0
Ear and labyrinth disorders	1 (25.0)	0	1 (20.0)	0	1 (16.7)	0	3 (20.0)	0
Tinnitus	0	0	0	0	1 (16.7)	0	1 (6.7)	0
Tympanic membrane perforation	1 (25.0)	0	0	0	0	0	1 (6.7)	0
Vertigo	0	0	1 (20.0)	0	0	0	1 (6.7)	0
Investigations	0	0	2 (40.0)	1 (20.0)	1 (16.7)	0	3 (20.0)	1 (6.7)
Alanine aminotransferase increased	0	0	1 (20.0)	0	1 (16.7)	0	2 (13.3)	0
Aspartate aminotransferase increased	0	0	1 (20.0)	0	0	0	1 (6.7)	0
Lipase increased	0	0	1 (20.0)	0	0	0	1 (6.7)	0
Weight decreased	0	0	1 (20.0)	1 (20.0)	0	0	1 (6.7)	1 (6.7)
Neoplasms benign, malignant and unspecified (incl cysts and polyps)	1 (25.0)	0	1 (20.0)	0	1 (16.7)	0	3 (20.0)	0
Cancer pain	0	0	1 (20.0)	0	0	0	1 (6.7)	0
Skin papilloma	0	0	0	0	1 (16.7)	0	1 (6.7)	0
Tumor-associated fever	1 (25.0)	0	0	0	0	0	1 (6.7)	0
Respiratory, thoracic and mediastinal disorders	1 (25.0)	0	2 (40.0)	0	0	0	3 (20.0)	0
Hiccups	0	0	1 (20.0)	0	0	0	1 (6.7)	0
Hypoxia	1 (25.0)	0	0	0	0	0	1 (6.7)	0
Oropharyngeal pain	0	0	1 (20.0)	0	0	0	1 (6.7)	0
Rhinorrhoea	0	0	1 (20.0)	0	0	0	1 (6.7)	0
Eye disorders	0	0	1 (20.0)	0	1 (16.7)	0	2 (13.3)	0
Conjunctival hemorrhage	0	0	0	0	1 (16.7)	0	1 (6.7)	0
Diplopia	0	0	1 (20.0)	0	0	0	1 (6.7)	0
Dry eye	0	0	0	0	1 (16.7)	0	1 (6.7)	0
Injury, poisoning and procedural complications	0	0	1 (20.0)	0	1 (16.7)	0	2 (13.3)	0
Nail avulsion	0	0	1 (20.0)	0	1 (16.7)	0	2 (13.3)	0
Psychiatric disorders	0	0	1 (20.0)	0	1 (16.7)	0	2 (13.3)	0
Insomnia	0	0	1 (20.0)	0	1 (16.7)	0	2 (13.3)	0
Anxiety	0	0	1 (20.0)	0	0	0	1 (6.7)	0
Cardiac disorders	0	0	0	0	1 (16.7)	0	1 (6.7)	0
Bundle branch block right	0	0	0	0	1 (16.7)	0	1 (6.7)	0
Endocrine disorders	1 (25.0)	0	0	0	0	0	1 (6.7)	0
Hypercalcemia of malignancy	1 (25.0)	0	0	0	0	0	1 (6.7)	0
Reproductive system and breast disorders	0	0	1 (20.0)	0	0	0	1 (6.7)	0
Prostatic obstruction	0	0	1 (20.0)	0	0	0	1 (6.7)	0

Treatment schedule: Cohort 1: 135 μg/kg SC QW, Cohort 2: 400 μg/kg SC QW, and Cohort 3: 800 μg/kg SC Q2W. Percentages in the total columns and toxicity grade columns are calculated with the number of patients in each treatment group as denominator

*COVID* coronavirus disease, *QW* every week, *Q2 W* every 2 weeks, *TEAE* treatment-emergent adverse events

Across all Cohorts, the most common TEAEs of any Grade were neutropenia (n = 9, 60.0%) followed by lymphopenia (n = 8, 53.3%), pyrexia and CRS (n = 7 each, 46.7%). The most frequently reported Grade 3 or 4 TEAEs across all Cohorts were neutropenia (n = 9, 60.0%), lymphopenia (n = 8, 53.3%), anemia (n = 5, 33.3%), and thrombocytopenia (n = 3, 20.0%). A total of 2 patients reported treatment-emergent SAEs of vertigo and gastroenteritis (n = 1, 6.7%, each), and vertigo was the only treatment-emergent SAEs related to the drug (n = 1, 6.7%). In all, 9 (60.0%) patients developed TEAEs leading to cycle delay or dose modification. No dose reductions, deaths or treatment discontinuation due to AEs were reported during the study.

The CRS events were not observed in Cohort 1. However, in Cohorts 2 and 3, a total of 7 patients experienced CRS events, all with a maximum toxicity of Grade 1 (7/15, 46.7% [Cohort 2 = 2/5; Cohort 3 = 5/6]). The median time to CRS onset relative to the most recent talquetamab dose was 1.2-days (range, 0.9–2.3), while median (range) duration of CRS was 1.5 days (0.1 to 9.6). CRS events occurred during step-up dose 1 (4/15; 26.7%), followed by step-up dose 2 (3/15; 20.0%), step-up dose 3 (2/15; 13.3%) and Cycle 1 day 1 (2/15; 13.3%). A total of 6 (40%) patients out of 15 received tocilizumab and no patient received steroid as supportive measures for CRS. All the CRS events were recovered or resolved during the study (Supplementary Table S2). Neurotoxicity (Grade 1 insomnia) was observed in only 1 patient (6.7%, Cohort 3 = 1/15) and no ICANS was reported.

Hematologic AEs were reported in 14 of 15 patients. Grade 3 or 4 hematologic AEs were reported in 13 of 15 patients (Table 2). Neutropenia (6/15, 40.0%), anemia (1/15, 6.7%) and thrombocytopenia (1/15, 6.7%) were the primary cause of cycle delay or dose modification in the study population.

Infections and infestations were observed in a total of 8/15 (53.3%) patients, with upper respiratory tract infections (13.3%) being the most frequent (Table 2). A COVID-19 infection in a patient from Cohort 2 (1/15, 6.7%) resulted in a delay in the treatment cycle or modification of dose.

Overall, talquetamab specific AEs were dysgeusia (including ageusia, dysgeusia, hypogeusia, and taste disorder); skin toxicity (including skin exfoliation, dry skin, pruritus, palmar-plantar erythrodysaesthesia syndrome); rash (including rash, rash maculo-papular, rash erythematous, erythema); nail disorders and were predominantly of Grade 1 or 2 in severity (Table 2). Dysgeusia related AEs were seen in 66.7% (n = 10) of patients, followed by skin toxicity in 33.3% (n = 5), rash in 20% (n = 3), nail disorders in 13.3% (n = 2) and dry mouth in 6.7% (n = 1) of patients. None of these AEs resulted in discontinuation, cycle delays or modification of talquetamab dosage during the study.

All dysgeusia events reported in 10 patients, were of Grade 1/2, (where grade 2 dysgeusia was reported only in 1 patient). The median time to onset of dysgeusia was 13.5 days. At the data-cutoff, 4 out of 10 dysgeusia events in 4 patients were recovered (Supplementary Table S3). Dysgeusia occurred both during step-up dosing and afterwards till Cycle 1 day 8. The medications used included benfotiamine, cyanocobalamin, and pyridoxine hydrochloride combination (1 [6.7%] patient) and zinc acetate (1 [6.7%] patient). The details of patients experiencing dysgeusia specific symptoms are presented in Supplementary Table S4.

Skin toxicities, including skin exfoliation, dry skin, pruritus, palmar-plantar erythrodysesthesia syndrome, were reported by approximately one-third of the patients (33.3%, 5/15). The median time to onset skin toxicities from the initial step-up dose was 20 days (range, 15–243). More than half of the events (4/7) were recovered by the data cut-off date (Supplementary Table S5; Supplementary Figure S1).

Rash, including maculo-papular, erythematous, erythema was reported in 3 of 15 patients (20.0%) with maximum toxicity of Grade 2 (Supplementary Table S6).

Nail disorders, including discoloration, onycholysis, onychomadesis, onychoclasis, dystrophy, toxicity, ridging were reported in 2 of the 15 patients (13.3%) with maximum toxicity of Grade 2 (Supplementary Figure S1). Half of the nail disorder events (2/4, 50.0%) were recovered at the data cut-off date.

One event of weight decrease (Grade 3) was reported by a patient (male, aged 60) in Cohort 2 (400 ug/kg QW); however, event was judged as not related to talquetamab by the investigator.

Alopecia was reported in 4 of 15 patients (Cohort 1 = 1/4 and Cohort 3 = 3/6). Most cases were of Grade 1, except for one patient in Cohort 3 (Grade 2). No apparent trend was observed in demographics of patients with alopecia.

### Pharmacokinetics

**Fig. 2:**
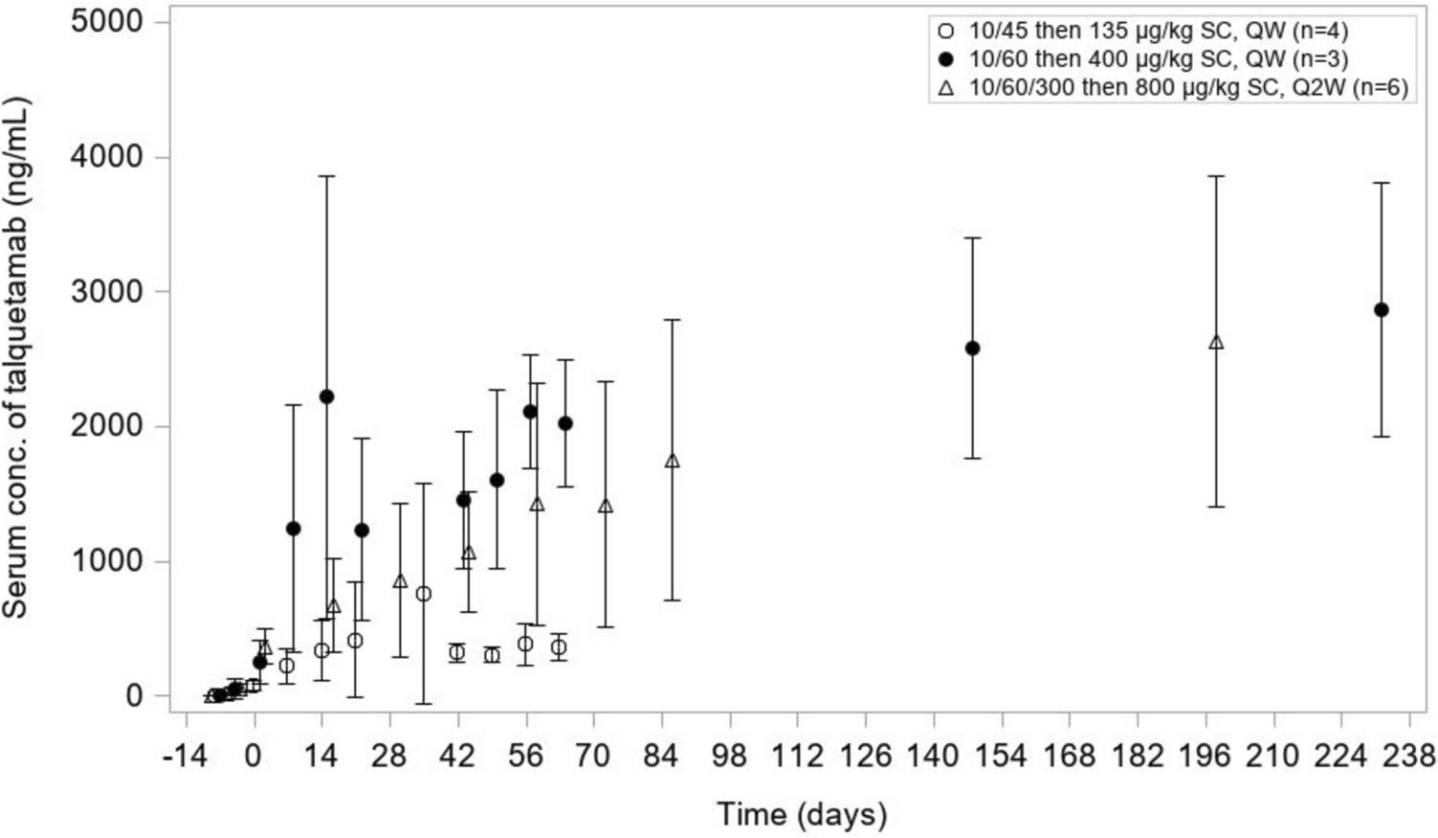
Linear mean (SD) pre-dose serum concentration–time profiles of talquetamab after SC administration of talquetamab. Treatments at 135 µg/kg weekly (with step-up doses of 10 and 45 µg/kg), at 400 µg/kg weekly (with step-up doses of 10 and 60 µg/kg), and at 800 µg/kg biweekly (with step-up doses of 10, 60 and 300 µg/kg)

Pre-dose serum concentration–time profiles of talquetamab after SC administration is presented in Fig. 2. Mean serum C_trough_ was higher than the maximum EC_90_ values (353 ng/mL) identified in an ex vivo cytotoxicity assay from first target dose of both weekly dosing of 400 µg/kg QW and biweekly dosing of 800 µg/kg Q2 W.

### Efficacy

The ORR (stringent complete response [sCR] + CR + very good partial response [VGPR] + PR) was 60.0% (9/15 patients; 95% CI 32.3%, 83.7%). In Cohort 1 the ORR was 25.0% (1/4 patients, 95% CI 0.6%, 80.6%), Cohort 2: 60.0% (3/5 patients, 95% CI 14.7%, 94.7%), and Cohort 3: 83.3% (5/6 patients, 95% CI 35.9%, 99.6%). VGPR or better response (sCR + CR + VGPR) was 26.7% (4/15 patients; 95% CI 7.8%, 55.1%). In Cohort 1 the VGPR or better response was achieved by none of the patients, Cohort 2: 20.0% (1/5 patients, 95% CI 0.5%, 71.6%), and Cohort 3: 50% (3/6 patients, 95% CI 11.8, 88.2) patients. The CR or better response (sCR + CR) was 13.3% (2/15 patients, 95% CI 1.7%, 40.5%). In Cohort 1 the CR or better response was achieved by none of the patients, Cohort 2: 20.0% (1/5 patients, 95% CI 0.5%, 71.6%), and Cohort 3: 16.7% (1/6 patients, 95% CI 0.4%, 64.1%) patients (Table 3).

**Table 3:** Response to talquetamab in patients treated with 2 RP2D and other doses

	Cohort 1 (n = 4)		Cohort 2 (n = 5)		Cohort 3 (n = 6)		Total (N = 15)	
	n (%)	95% CI	n (%)	95% CI	n (%)	95% CI	n (%)	95% CI
Response category								
Stringent complete response (sCR)	0	(NE, NE)	0	(NE, NE)	1 (16.7)	(0.4, 64.1)	1 (6.7)	(0.2, 31.9)
Complete response (CR)	0	(NE, NE)	1 (20.0)	(0.5, 71.6)	0	(NE, NE)	1 (6.7)	(0.2, 31.9)
Very good partial response (VGPR)	0	(NE, NE)	0	(NE, NE)	2 (33.3)	(4.3, 77.7)	2 (13.3)	(1.7, 40.5)
Partial response (PR)	1 (25.0)	(0.6, 80.6)	2 (40.0)	(5.3, 85.3)	2 (33.3)	(4.3, 77.7)	5 (33.3)	(11.8, 61.6)
Minimal response (MR)	0	(NE, NE)	0	(NE, NE)	1 (16.7)	(0.4, 64.1)	1 (6.7)	(0.2, 31.9)
Stable disease (SD)	2 (50.0)	(6.8, 93.2)	0	(NE, NE)	0	(NE, NE)	2 (13.3)	(1.7, 40.5)
Progressive disease (PD)	1 (25.0)	(0.6, 80.6)	1 (20.0)	(0.5, 71.6)	0	(NE, NE)	2 (13.3)	(1.7, 40.5)
Overall response (sCR + CR + VGPR + PR)	1 (25.0)	(0.6, 80.6)	3 (60.0)	(14.7, 94.7)	5 (83.3)	(35.9, 99.6)	9 (60.0)	(32.3, 83.7)
VGPR or better (sCR + CR + VGPR)	0	(NE, NE)	1 (20.0)	(0.5, 71.6)	3 (50.0)	(11.8, 88.2)	4 (26.7)	(7.8, 55.1)
CR or better (sCR + CR)	0	(NE, NE)	1 (20.0)	(0.5, 71.6)	1 (16.7)	(0.4, 64.1)	2 (13.3)	(1.7, 40.5)

Time to response (months)*	Median	Range	Median	Range	Median	Range	Median	Range
Time to first response (PR or better)	1.68	(1.7–1.7)	1.05	(0.9–1.2)	1.38	(1.2–2.2)	1.31	(0.9–2.2)
Time to best response	1.68	(1.7–1.7)	1.22	(0.9–13.0)	6.87	(1.4–12.8)	2.23	(0.9–13.0)
Time to VGPR or better	–	–	7.26	(7.3–7.3)	6.87	(4.0–7.0)	6.95	(4.0–7.3)
Time to CR or better	–	–	12.98	(13.0–13.0)	12.78	(12.8–12.8)	12.88	(12.8–13.0)
Duration of response (Median [95% CI])	6.5	(NE–NE)	14.3	(7.6–NE)	7.5	(2.8–NE)	7.6	(2.8–NE)

^*^For time to response and DOR, Cohort 1 N = 1; Cohort 2 N = 3; Cohort 3 N = 5; Treatment schedule: Cohort 1: 135 μg/kg SC QW, Cohort 2: 400 μg/kg SC QW, and Cohort 3: 800 μg/kg SC Q2 W

*CI* confidence interval, *CR* complete response, *DOR* duration of response, *MR* minimal response, *NE* not estimable, *PR* partial response, *PD* progressive disease, *sCR* stringent complete response, *QW* every week, *Q2 W* every 2 weeks, *SD* stable disease, *VGPR* very good partial response

The overall, median duration of follow-up among responders (n = 9), was 14.62 months (range, 6.8–22.0). The overall median DOR was 7.6 months (range, 2.8–NE) (Table 3). The probability of responders remaining in response at 6-months was 77.8% (95% CI 36.5%, 93.9%) while it was 44.4% (95% CI 13.6, 71.9) at months 9 and 12. In Cohort 1, 2 and 3 the median DOR was 6.5 months, 14.3 months, and 7.5 months respectively.

**Fig. 3:**
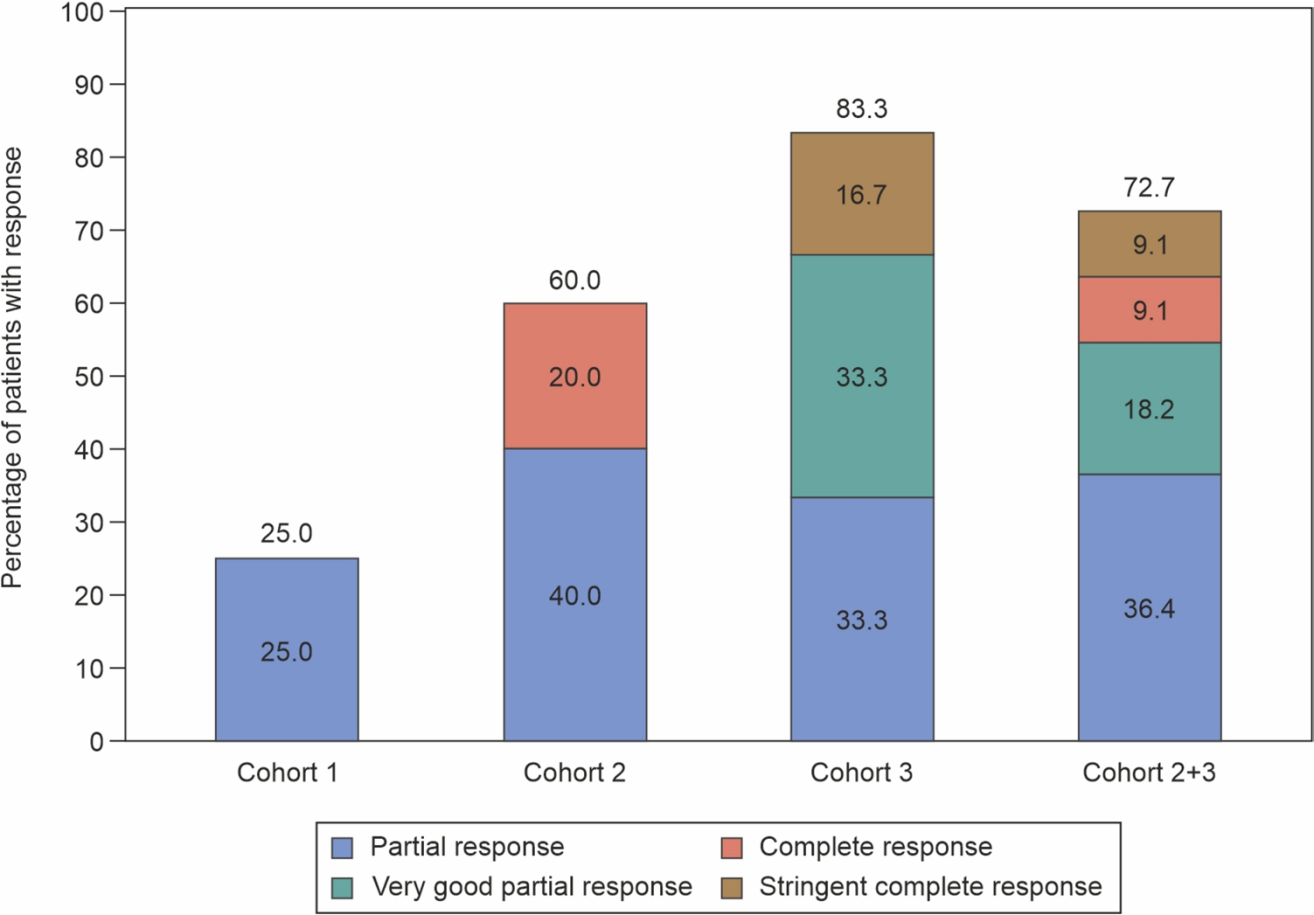
Response to talquetamab therapy in patients with RRMM. Treatments: at Cohort 1, 135 µg/kg Weekly (with step-up doses of 10 and 45 µg/kg), at cohort 2, 400 µg/kg weekly (with step-up doses of 10 and 60 µg/kg), and at cohort 3, 800 mg/kg biweekly (with step-up doses of 10, 60 and 300 µg/kg). Response was assessed by investigator, based on International Myeloma Working Group (IMWG) consensus criteria (2016)

**Fig. 4:**
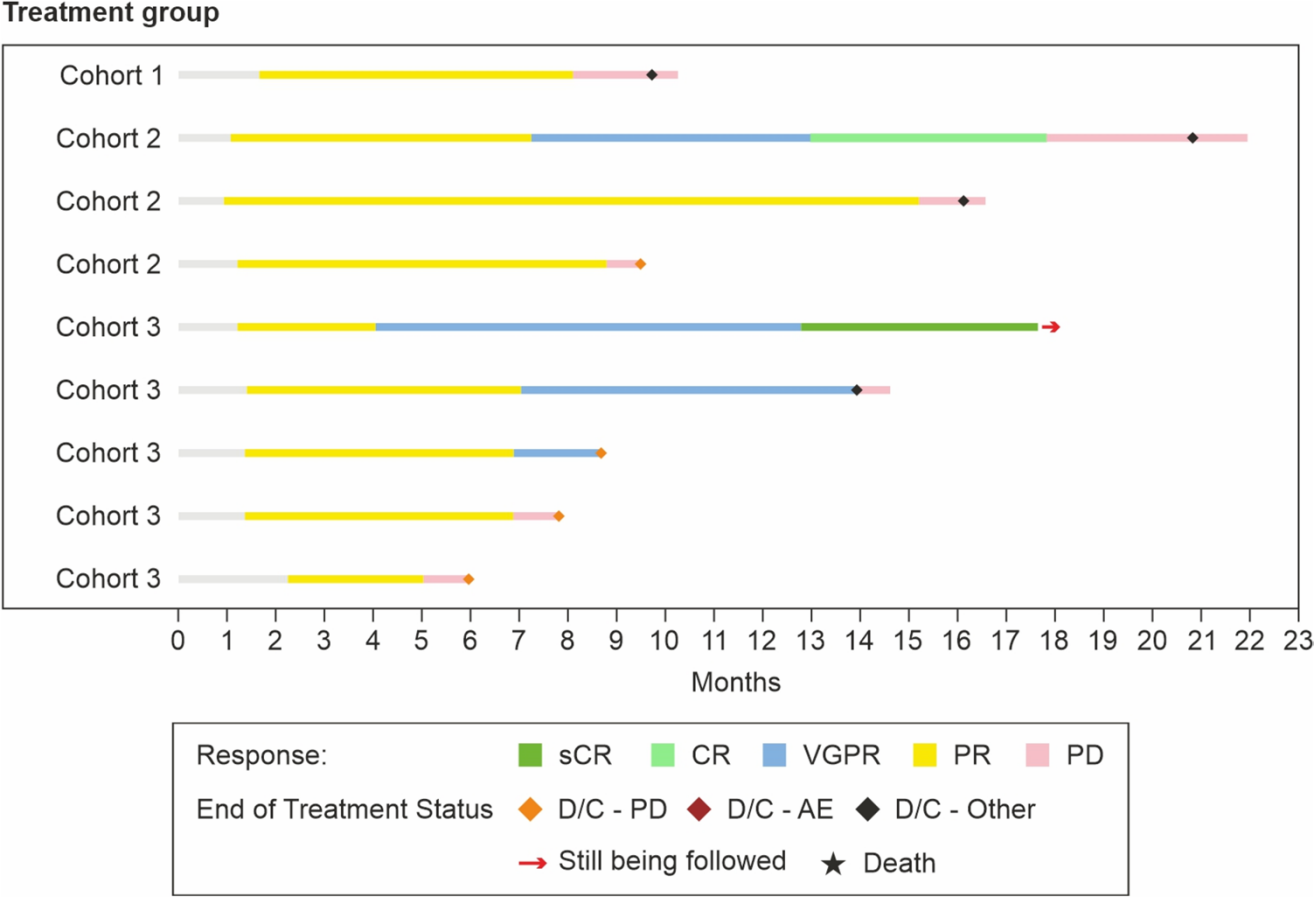
Responses over time in patients who received talquetamab. Treatments: at cohort 1, 135 µg/kg weekly (with step-up doses of 10 and 45 µg/kg), at cohort 2, 400 µg/kg weekly (with step-up doses of 10 and 60 µg/kg), and at cohort 3, 800 mg/kg biweekly (with step-up doses of 10, 60 and 300 µg/kg). Response was assessed by investigator, based on International Myeloma Working Group (IMWG) consensus criteria (2016). *AE* adverse events, *CR* complete response, *D/C* discontinued, *PD* progressive disease, *PR* partial response, *sCR* stringent complete response, *VGPR* very good partial response

Overall, the median time to response was 1.3 months (range, 0.9–2.2) for first response (PR or better), 2.2 months (range, 0.9–13.0) for best response, 7.0 months (range, 4.0–7.3) for VGPR or better, and 12.9 months (range, 12.8–13.0) for CR or better response. In Cohorts 1, 2 and 3 the median time to best response was 1.7 months, 1.2 months, and 6.9 months respectively) (Fig. 3; Table 3). The cohort-wise responses over time in patients receiving talquetamab is shown in Fig. 4.

There were 4 patients with double high-risk cytogenetic factor at baseline. Cohort 2 had one patient with del(17p), t(4;14). Cohort 3 had 3 patients: 2 patients with del(17p), 1q gain and 1 patient with t(4;14), 1q gain. The best response in these patients was a VGPR in patient with t(4:14) and 1q gain and a PR in patients with del(17p) and 1q gain and del(17p), t(4;14).

## Discussion

In this phase 1 study involving heavily pretreated RRMM Japanese patients, treatment with the 2 RP2Ds identified in the global, pivotal, phase 1/2 MonumenTAL-1 study, resulted in an acceptable safety profile with no new safety signals in Japanese population. With no DLT effects observed, the current study revealed a comparable safety profile to the global population where AEs were marked by T cell activation (e.g., CRS and GPRC5D associated [22]). All CRS events were of Grade 1 and well managed with supportive care including tocilizumab. Only 1 Grade 1 neurotoxicity was observed without ICANS across the 3 Cohorts (Cohort 1: 135 μg/kg SC QW, Cohort 2: 400 μg/kg SC QW, and Cohort 3: 800 μg/kg SC Q2 W). Dysgeusia was seen as the most frequently observed treatment-related toxicity. Although 60% of dysgeusia cases remained unresolved during the study, patients continued their talquetamab treatment without any dose modification or treatment discontinuation and therefore, dysgeusia was clinically manageable. Moreover, 40% of patients recovered from dysgeusia, with an overall median duration of 309.0 days (range, 195–481), suggesting that the dysgeusia induced by talquetamab treatment was reversible. Most cases of dysgeusia did not coincide with appetite loss or weight loss. All the other treatment-related toxicities (skin, dry mouth, rash, and nail disorders) were also of low Grade (maximum Grade 2) and were well managed without any dose modification or treatment discontinuation.

The AEs observed with talquetamab, such as dysgeusia, skin disorders, dry mouth, rash, and nail toxicities, align with its mechanism as a GPRC5D-targeting bispecific antibody. Dysgeusia and nail toxicities likely result from on-target, off-tumor effects, as GPRC5D is expressed in filiform papillae (lacking taste receptors) and residential plasma cells in the oral cavity [22], as well as in the nail keratogenous zone in preclinical models [23]. GPRC5D is also expressed in skin structures like hair follicles and eccrine glands [22], though the exact mechanisms driving skin, nail, and oral AEs remain unclear [24].

Talquetamab was well tolerated at the RP2D with no AE related treatment discontinuation or dose reduction seen in Japanese population [25]. Moreover, the overall, safety profile of talquetamab in Japanese RRMM patients was similar to those reported in non-Japanese global population in MonumenTAL-1 and those reported with other GPRC5D targeted T cell-redirecting therapies as well [26–28].

The Japanese patients treated with the 2 RP2D of talquetamab (Cohort 2-400 μg/kg SC QW, and Cohort 3: 800 μg/kg SC Q2 W) showed a notable response to treatment which continued over time and was similar to the global phase 1/2 patient population in the MonumenTAL-1 study [25]. Although a direct comparison between the studies for response rates is not possible, talquetamab yielded a comparable ORR to other novel approved MM therapies in similar patient populations in Japan [29, 30]. Furthermore, a comparable ORR to other published T cell-redirecting BsAb GPRC5D/CD3 (forimtamig) and other recently published GPRC5D CAR-T therapies was observed [26–28, 31].

The current study found that one patient—who had previously undergone BCMA CAR-T cell therapy and was treated with the RP2D of talquetamab, achieved a CR to talquetamab with a DOR lasting 16.8-months. This observation further aligns with the finding from global MonumenTAL-1 [32] and studies on other GPRC5D CAR-T cell therapies, [26, 33] suggesting that targeting a unique antigen expression like GPRC5D could possibly be a treatment choice for patients who received anti-BCMA target treatment in Japanese as well as non-Japanese populations.

Despite an overall good response rate observed in Cohort 3 (receiving 800 μg/kg SC Q2 W), the median duration of response to talquetamab was relatively short (7.5 months, 95% CI 2.8, NE) in this Cohort. This may be attributed to the background in 3 patients whose cytogenetics findings had a double high-risk cytogenetic factor at baseline, but the sample size was small in this study and further detailed analysis would be required to confirm this relationship.

A few limitations of this study need to be considered when interpreting the results. The number of patients in each dosage Cohorts was small, and the single-arm nature of the study makes it difficult to make direct comparisons of the results with the approved or investigational agents. To confirm the findings of this study, further evaluation in a larger number of Japanese patients with longer follow-up time would be required. The Japanese phase 2 study is ongoing as a part of global MonumenTAL-1 to assess efficacy and safety of talquetamab including quality of life (QOL) assessments in Japanese population with a larger sample size.

## Conclusion

In conclusion, with a manageable safety profile talquetamab showed favorable efficacy results in treating Japanese patients with triple-class exposed RRMM who have received multiple lines of therapy. Although the number of Japanese patients included in this study was small, the safety and efficacy findings are quite valuable and aligned with those of the global population in MonumenTAL-1 study. Also, current results suggest that Japanese patients with RRMM previously refractory to BCMA CAR-T cell therapy might benefit from talquetamab. However, owing to a small number of Japanese patient population in this study, further clinical data are needed to demonstrate and validate the safety and efficacy of talquetamab in Japanese population.

## Supplementary Information

Below is the link to the electronic supplementary material.

Supplementary file1 (DOCX 1577 KB)

## Data Availability

Although the current study data are not currently available for sharing, requests can be sent to the corresponding author and will be evaluated on an individual basis.
