# Supplementary material for: Phase 1 study of talquetamab, a humanized GPRC5D x CD3 bispecific antibody, in Japanese patients with relapsed/refractory MM
Source: Int J Hematol. 2025 May 9;122(3):421–33. doi: 10.1007/s12185-025-03991-5 (PMC12380926; doi:10.1007/s12185-025-03991-5)
Supplement: Supplementary file 1 — Supplementary file1 (DOCX 1577 KB) [file 12185_2025_3991_MOESM1_ESM.docx]

**Supplementary Information**

**Supplementary Figure S1**: Characteristic pictures for skin and nail disorders related to talquetamab

Treatments: at Cohort 1, 135 µg/kg Weekly (with Step-up Doses of 10 and 45 µg/kg), at Cohort 2, 400 µg/kg Weekly (with Step-up Doses of 10 and 60 µg/kg), and at Cohort 3, 800 mg/kg Biweekly (with Step‑up Doses of 10, 60, and 300 µg/kg).

QW, every week; Q2W, every 2 weeks.

**Supplementary Table S1** Dose-limiting toxicity criteria^a^

^a^The SET will evaluate all TLS events and decide if the circumstances meet the criteria for DLT. ^b^If the AE is part of a syndrome (eg, TLS, CRS, or sARR), then the grade of the syndrome should dictate the DLT assessment. ^c^Hy’s Law criteria, defined as ALT or AST value ≥3 x ULN, total bilirubin ≥2 x ULN, and alkaline phosphatase ≤2 x ULN; with no alternative etiology. ^d^More frequent laboratory monitoring including CBC and differential should be initiated to document the start and the resolution of the specific toxicity; persistence needs to be documented by a CBC with differential blood count immediately after the time limitation for the specific toxicity has expired. ^e^HTN occurring as part of a CRS event should be considered as a symptom of CRS, in which case the grade of the syndrome should dictate the DLT assessment. ^f^Regarding study treatment related AEs which do not meet DLT definition, but the subject misses at least 2 planned doses, or has dosing interrupted for >28 days from last administration during DLT evaluation period, such AEs will be regarded as a DLT.

AE, adverse event; ALT, alanine aminotransferase; AST, aspartate aminotransferase; CBC, complete blood count; CRS, cytokine release syndrome; DLT, dose-limiting toxicity; hrs, hours; sARR, systemic administration-related reactions; SET, Study Evaluation Team; TLS, tumor lysis syndrome; ULN, upper limit of normal.

**Supplementary Table S2**: Summary of treatment-emergent CRS events (All treated analysis set)

Treatment schedule: Cohort 1: 135 μg/kg SC QW, Cohort 2: 400 μg/kg SC QW, and Cohort 3: 800 μg/kg SC Q2W.

Data presented in n (%) unless indicated. ^a^CRS events are evaluated according to ASTCT (Lee 2019) consensus grading system. ^b^Supportive measures to treat CRS and CRS symptoms are included. ^c^Other supportive measures to treat CRS include MOHRUS PAPS XR. Percentages calculated with the number of patients in all treated analysis set as denominator, except for Number of CRS Events and outcome of CRS for which percentages are calculated with the number.

CRS, Cytokine Release Syndrome; IL, interleukin; IV, intravenous; QW, every week; Q2W, every 2 weeks.

**Supplementary Table S3**: Summary of treatment-emergent adverse events of clinical interest of dysgeusia (All treated analysis set)

Treatment schedule: Cohort 1: 135 μg/kg SC QW, Cohort 2: 400 μg/kg SC QW, and Cohort 3: 800 μg/kg SC Q2W. Data presented in n (%) unless indicated. *Including ageusia, dysgeusia, hypogeusia, and taste disorder. ^a^Patients may appear in more than one category. ^b^Occurrence is based on the last treatment visit on or prior to the day in which the TEAE occurred. ^c^Include AEs with both start and end dates are available. ^d^Concurrent events considers events that occur during or within 30 days of the end date of Dysgeusia*. ^e^Weight decrease is defined as weight decreases at least 10% (>=10%) from baseline recorded in the vital signs. Percentages calculated with the number of patients in the All Treated Analysis Set as denominator, except for the concurrent events for which percentages are calculated with the number of patients with Dysgeusia* in the All Treated Analysis Set as denominator and outcome of Dysgeusia* for which percentages are calculated with the number of Dysgeusia* events in the All Treated Analysis Set as denominator.

QW, every week; Q2W, every 2 weeks; TEAE, treatment-emergent adverse event.

**Supplementary Table S4**: Patient demographic and detail symptoms for dysgeusia

Treatments: Cohort 1: 135 µg/kg QW; Cohort 2: 400 µg/kg QW; Cohort 3: 800 µg/kg Q2W.

QW, every week; Q2W, every 2 weeks.

**Supplementary Table S5**: Summary of treatment-emergent adverse events of clinical interest of skin toxicity (All treated analysis set)

*Including Skin exfoliation, Dryskin,Pruritus, Palmar-plantar erythrodysaesthesia syndrome.

Treatment schedule: Cohort 1: 135 μg/kg SC QW, Cohort 2: 400 μg/kg SC QW, and Cohort 3: 800 μg/kg SC Q2W. All concomitant medications for Skin Toxicity* are included. ^a^Patients may appear in more than one category. ^b^Occurrence is based on the last treatment visit on or prior to the day in which the TEAE occurred. ^c^AEs with both start and end dates are available. ^d^All concomitant medications for Skin Toxicity* are included. Percentages calculated with the number of patients in the All Treated Analysis Set as denominator, except for the outcome of Skin Toxicity*.

QW, every week; Q2W, every 2 weeks; TEAE, treatment-emergent adverse event.

**Supplementary Table S6**: Summary of treatment-emergent adverse events of clinical interest of rash (All treated analysis set)

Treatment schedule: Cohort 1: 135 μg/kg SC QW, Cohort 2: 400 μg/kg SC QW, and Cohort 3: 800 μg/kg SC Q2W. *including rash, rash maculo-papular, rash erythematous, erythema. ^a^Patients may appear in more than one category. ^b^Occurrence is based on the last treatment visit on or prior to the day in which the TEAE occurred. ^c^Includes AEs with both start and end dates are available. ^d^All concomitant medications for Rash* are included. Percentages calculated with the number of patients in all treatment analysis set as denominator, except for the outcome of Rash*.

NOTE: For Nail disorders only one patient (1 [16.7%]) was given the concomitant medication (HEPARINOID) in Cohort 3.

QW, every week; Q2W, every 2 weeks; TEAE, treatment-emergent adverse event.

**Supplementary Table S7:** Patient prior CAR-T cell therapies and response status to talquetamab

^a^Study day is relative to the date of first step-up dose of study agent. Treatments: Cohort 1: 135 µg/kg QW; Cohort 2: 400 µg/kg QW; Cohort 3: 800 µg/kg Q2W.

BCMA, B-cell maturation antigen; CAR-T, Chimeric Antigen Receptor T-cell therapy; QW, every week; Q2W, every 2 weeks.


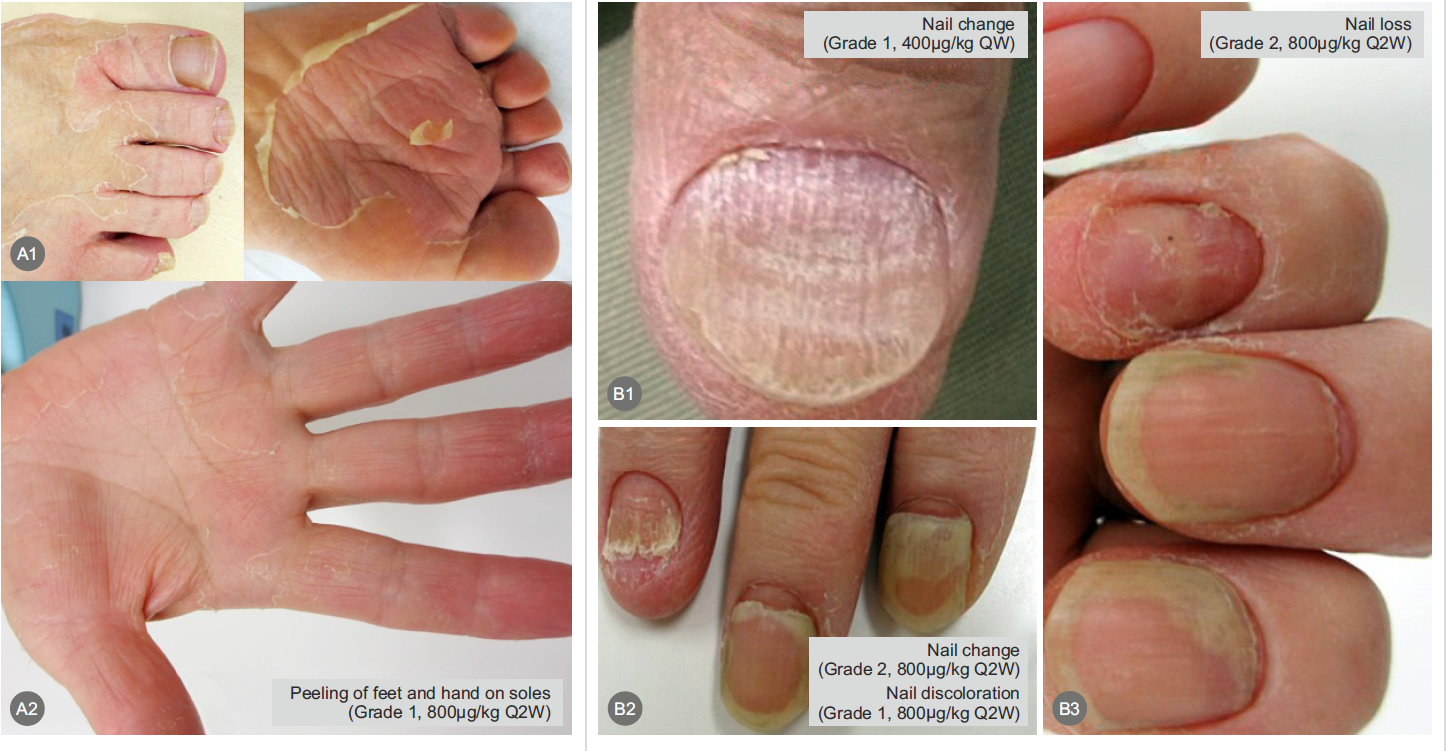
**Figure S1**: Characteristic pictures for skin (A1–A2) and nail disorders (B1–B3) related to talquetamab

Treatments: at Cohort 1, 135 µg/kg Weekly (with Step-up Doses of 10 and 45 µg/kg), at Cohort 2, 400 µg/kg Weekly (with Step-up Doses of 10 and 60 µg/kg), and at Cohort 3, 800 mg/kg Biweekly (with Step‑up Doses of 10, 60 and 300 µg/kg).

QW, every week; Q2W, every 2 weeks.

**Supplementary Table S1** Dose-limiting toxicity criteria^a^

| **Non-hematological toxicity** | |
| --- | --- |
| TLS^a,b^ | Grade ≥3 with clinical sequelae that does not resolve within 72 hrs |
| Other non-hematological toxicity^b^ | Grade ≥3, except:   - Grade ≥3 sARR or CRS that resolves to Grade ≤1 within 48 hrs - Grade 3 asthenia, fever, or constipation - Grade 3 nausea, vomiting, diarrhea, or pain that resolve within 72 hrs - Grade 3 hypertension that resolved in ≤4 hrs^e^ - First occurrence of Grade 3 CRS - First occurrence of Grade 3 rash that improves to ≤Grade 2 within 7 days with appropriate treatment, including oral   steroids |
| **Clinical laboratory abnormalities** | |
| AST or ALT^b^ | - Grade 3 unless resolved to Grade ≤1 (or baseline) within 72 hrs - Grade 4 - Meeting criteria for Hy’s law^c^ |
| Lipase or amylase^b^ | Grade ≥3 associated with clinical or radiological evidence of pancreatitis |
| Other chemistry abnormalities^b^ | - Grade ≥3 and associated with clinical complications unless resolved to Grade ≤1 (or baseline) within 72 hrs - Grade 4 unless resolved within 24 hours to Grade ≤1 or baseline or related to CRS or infusion-related event - Grade 5 |
| **Hematological toxicity** | |
| Neutrophil count decreased^b^ | Grade 4 for >7 days^d^ |
| Febrile neutropenia | - Grade 3 that does not resolve within 7 days - Grade 4 |
| Platelet count decreased^b^ | - Grade 3 with clinically significant bleeding or the requirement for platelet transfusion - Grade 4 |
| Any hematological toxicity | Grade 5 |

^a^The SET will evaluate all TLS events and decide if the circumstances meet the criteria for DLT. ^b^If the AE is part of a syndrome (eg, TLS, CRS, or sARR), then the grade of the syndrome should dictate the DLT assessment. ^c^Hy’s Law criteria, defined as ALT or AST value ≥3 x ULN, total bilirubin ≥2 x ULN, and alkaline phosphatase ≤2 x ULN; with no alternative etiology. ^d^More frequent laboratory monitoring including CBC and differential should be initiated to document the start and the resolution of the specific toxicity; persistence needs to be documented by a CBC with differential blood count immediately after the time limitation for the specific toxicity has expired. ^e^HTN occurring as part of a CRS event should be considered as a symptom of CRS, in which case the grade of the syndrome should dictate the DLT assessment. ^f^Regarding study treatment related AEs which do not meet DLT definition, but the subject misses at least 2 planned doses, or has dosing interrupted for >28 days from last administration during DLT evaluation period, such AEs will be regarded as a DLT.

AE, adverse event; ALT, alanine aminotransferase; AST, aspartate aminotransferase; CBC, complete blood count; CRS, cytokine release syndrome; DLT, dose-limiting toxicity; hrs, hours; sARR, systemic administration-related reactions; SET, Study Evaluation Team; TLS, tumor lysis syndrome; ULN, upper limit of normal.

**Supplementary Table S2**: Summary of treatment-emergent CRS events (All treated analysis set)

|  | **Cohort 1 (n=4)** | **Cohort 2 (n=5)** | **Cohort 3 (n=6)** | **Total (N=15)** |
| --- | --- | --- | --- | --- |
| **Patients with CRS, n (%)** | 0 | 2  (40.0) | 5  (83.3) | 7  (46.7) |
| **Maximum toxicity grade^a^** | | | | |
| Grade 1 | 0 | 2  (40.0) | 5  (83.3) | 7  (46.7) |
| Grade 2 | 0 | 0 | 0 | 0 |
| Grade 3 | 0 | 0 | 0 | 0 |
| Grade 4 | 0 | 0 | 0 | 0 |
| Grade 5 | 0 | 0 | 0 | 0 |
| **Patients with multiple CRS events, n (%)** | 0 | 1  (20.0) | 1  (16.7) | 2  (13.3) |
| Grade of CRS worsened at any subsequent event | 0 | 0 | 0 | 0 |
| **CRS occurrence, Event Onset Time** | | | | |
| Step-up Dose 1 | 0 | 2  (40.0) | 2  (33.3) | 4  (26.7) |
| Step-up Dose 2 | 0 | 0 | 3  (50.0) | 3  (20.0) |
| Step-up Dose 3 | - | - | 2  (33.3) | 2  (13.3) |
| Repeat Step-up | 0 | 0 | 0 | 0 |
| Cycle 1 Day 1 | 0 | 1  (20.0) | 1  (16.7) | 2  (13.3) |
| Cycle 1 Day 8 | 0 | 0 | 0 | 0 |
| **Time from most recent dose to new onset of CRS, Median (range)** | 0 | 1.2  (0.9–1.5) | 1.2  (0.9–2.3) | 1.2  (0.9–2.3) |
| **Duration of CRS, days Median (range)** | 0 | 1.9  (0.7–9.6) | 1.1  (0.1–3.5) | 1.5  (0.1–9.6) |
| **Number of patients with supportive measures to treat CRS^b^, n (%)** | 0 | 2 | 5 | 7 |
| Anti-IL6 receptor tocilizumab | 0 | 2  (40.0) | 4  (66.7) | 6  (40.0) |
| Multiple doses at any time during study | 0 | 1  (20.0) | 1  (16.7) | 2  (13.3) |
| >1 dose for a single CRS event | 0 | 0 | 0 | 0 |
| Corticosteroids | 0 | 0 | 0 | 0 |
| IV Fluids | 0 | 0 | 0 | 0 |
| Vasopressor used | 0 | 0 | 0 | 0 |
| Oxygen used | 0 | 0 | 0 | 0 |
| Paracetamol | 0 | 2  (40.0) | 5  (83.3) | 7  (46.7) |
| Other^c^ | 0 | 1  (20.0) | 0 | 1  (6.7) |
| **Outcome of CRS events, n (%)** | | | | |
| Number of CRS events | 0 | 3 | 8 | 11 |
| Recovered/resolved | 0 | 3  (100.0) | 8  (100.0) | 11  (100.0) |
| Not recovered or not resolved | 0 | 0 | 0 | 0 |
| Recovered or resolved with sequelae | 0 | 0 | 0 | 0 |
| Recovering or resolving | 0 | 0 | 0 | 0 |
| Fatal | 0 | 0 | 0 | 0 |
| Unknown | 0 | 0 | 0 | 0 |
| Missing | 0 | 0 | 0 | 0 |

Treatment schedule: Cohort 1: 135 μg/kg SC QW, Cohort 2: 400 μg/kg SC QW, and Cohort 3: 800 μg/kg SC Q2W.

Data presented in n (%) unless indicated. ^a^CRS events are evaluated according to ASTCT (Lee 2019) consensus grading system. ^b^Supportive measures to treat CRS and CRS symptoms are included. ^c^Other supportive measures to treat CRS include MOHRUS PAPS XR. Percentages calculated with the number of patients in all treated analysis set as denominator, except for Number of CRS Events and outcome of CRS for which percentages are calculated with the number.

CRS, Cytokine Release Syndrome; IL, interleukin; IV, intravenous; QW, every week; Q2W, every 2 weeks.

**Supplementary Table S3**: Summary of treatment-emergent adverse events of clinical interest of dysgeusia (All treated analysis set)

|  | **Cohort 1 (n=4)** | | **Cohort 2 (n=5)** | **Cohort 3 (n=6)** | **Total (N=15)** |
| --- | --- | --- | --- | --- | --- |
| **Patients with dysgeusia n (%)*** | 3  (75.0) | 3  (60.0) | | 4  (66.7) | 10  (66.7) |
| **Maximum toxicity grade n (%)** | | | | | |
| Grade 1 | 3  (75.0) | 3  (60.0) | | 3  (50.0) | 9  (60.0) |
| Grade 2 | 0 | 0 | | 1  (16.7) | 1  (6.7) |
| Grade 3 | 0 | 0 | | 0 | 0 |
| Grade 4 | 0 | 0 | | 0 | 0 |
| Grade 5 | 0 | 0 | | 0 | 0 |
| **Number of patients with Dysgeusia* leading to discontinuation of study drug** | 0 | 0 | | 0 | 0 |
| **Number of patients with Dysgeusia* leading to dose modification^a^** | | | | | |
| Delayed | 0 | 0 | | 0 | 0 |
| Skipped | 0 | 0 | | 0 | 0 |
| Reduced | 0 | 0 | | 0 | 0 |
| **Occurrence of Dysgeusia*^a,b^ n (%)** |  |  | |  |  |
| Step-up Dose 1 | 0 | 0 | | 1  (16.7) | 1  (6.7) |
| Step-up Dose 2 | 0 | 1  (20.0) | | 1  (16.7) | 2  (13.3) |
| Step-up Dose 3 | - | - | | 0 | 0 |
| Repeat Step-up | 0 | 0 | | 0 | 0 |
| Cycle 1 Day 1 | 1  (25.0) | 2  (40.0) | | 2  (33.3) | 5  (33.3) |
| Cycle 1 Day 8 | 2  (50.0) | 0 | | 0 | 2  (13.3) |
| Subsequent Doses | 0 | 0 | | 0 | 0 |
| **Day of Dysgeusia* onset relative to initial step-up dose**  **Median (range)** | 19  (18–29) | 12  (11–12) | | 10.5  (3–16) | 13.5  (3–29) |
| **Duration of Dysgeusia*, days^c^ Median (range)** | 239.5  (195–284) | 481.0  (481–481) | | 334.0  (334–334) | 309.0  (195–481) |
| **Patients with Concomitant medications for Dysgeusia^*a,d^ n (%)** | 0 | 1  (20.0) | | 1  (16.7) | 2  (13.3) |
| Benfotiamine, cyanocobalamin, pyridoxine hydrochloride | 0 | 0 | | 1  (16.7) | 1  (6.7) |
| Zinc acetate | 0 | 1  (20) | | 0 | 1  (6.7) |
| **Concurrent decreased appetite^e^ n (%)** | | | | | |
| Yes | 0 | 0 | | 1  (25) | 1  (10) |
| No | 3  (100) | 3  (100) | | 3  (75) | 9  (90) |
| **Concurrent dry mouth^e^** | | | | | |
| Yes | 0 | 1  (33.3) | | 0 | 1  (10) |
| No | 3 (100) | 2  (66.7) | | 4  (100) | 9  (90) |
| **Concurrent weight decreased (TEAE)^e^** | | | | | |
| Yes | 0 | 0 | | 0 | 0 |
| No | 3  (100) | 3  (100) | | 4  (100) | 10  (100) |
| **Concurrent weight decreased (Vital signs)^e,f^** | | | | | |
| Yes | 0 | 0 | | 0 | 0 |
| No | 3  (100) | 3  (100) | | 4  (100) | 10  (100) |
| **Outcome of Dysgeusia*** |  |  | |  |  |
| Number of events | 3 | 3 | | 4 | 10 |
| Recovered or resolved | 2  (66.7) | 1  (33.3) | | 1  (25) | 4  (40) |
| Not recovered or not resolved | 1  (33.3) | 2  (66.7) | | 3  (75) | 6  (60) |
| Recovered or resolved with sequelae | 0 | 0 | | 0 | 0 |
| Recovering or resolving | 0 | 0 | | 0 | 0 |
| Fatal | 0 | 0 | | 0 | 0 |
| Unknown | 0 | 0 | | 0 | 0 |
| Missing | 0 | 0 | | 0 | 0 |

Treatment schedule: Cohort 1: 135 μg/kg SC QW, Cohort 2: 400 μg/kg SC QW, and Cohort 3: 800 μg/kg SC Q2W. Data presented in n (%) unless indicated. *Including ageusia, dysgeusia, hypogeusia, and taste disorder. ^a^Patients may appear in more than one category. ^b^Occurrence is based on the last treatment visit on or prior to the day in which the TEAE occurred. ^c^AEs with both start and end dates are available. ^d^All concomitant medications for Dysgeusia^*^ are included. ^e^Concurrent events considers events that occur during or within 30 days of the end date of Dysgeusia^*^. ^f^Weight decrease is defined as weight decreases at least 10% (>=10%) from baseline recorded in the vital signs. Percentages calculated with the number of patients in the all treated analysis set as denominator, except for the concurrent events for which percentages are calculated with the number of patients with Dysgeusia^*^ in the All Treated Analysis Set as denominator and outcome of Dysgeusia^*^ for which percentages are calculated with the number of Dysgeusia^*^ events in the All Treated Analysis Set as denominator.

QW, every week; Q2W, every 2 weeks; TEAE, treatment-emergent adverse event.

| **Treatment Cohort** | **Case** | **Onset**  **(day)** | **Grade** | **Reported specific symptoms** |
| --- | --- | --- | --- | --- |
| Cohort 1 | 1 | 29 | 1 | Diminished sense of taste |
|  | 2 | 19 | 1 | Diminished of taste (Saltiness) |
|  | 3 | 18 | 1 | Dissociated hypogeusia (Saltiness, Sweetness) |
| Cohort 2 | 4 | 12 | 1 | Diminished sense of taste |
|  | 5 | 11 | 1 | Loss of taste |
|  | 6 | 12 | 1 | Not reported |
| Cohort 3 | 7 | 3 | 1 | Diminished sense of taste |
|  | 8 | 6 | 2 | Heterogeusia  Dissociated hypogeusia (Sourness, Sweetness) |
|  | 9 | 15 | 1 | Dissociated hypogeusia (Sourness, Sweetness) |
|  | 10 | 16 | 1 | Dissociated dysgeusia (Sweetness)  Hypergeusia (Bitterness) |

**Supplementary Table S4**: Patient demographic and detail symptoms for dysgeusia

Treatments: Cohort 1: 135 µg/kg QW; Cohort 2: 400 µg/kg QW; Cohort 3: 800 µg/kg Q2W.

QW, every week; Q2W, every 2 weeks.

**Supplementary Table S5**: Summary of treatment-emergent adverse events of clinical interest of skin toxicity (All treated analysis set)

|  | | **Cohort 1 (n=4)** | **Cohort 2 (n=5)** | **Cohort 3 (n=6)** | **Total (N=15)** |
| --- | --- | --- | --- | --- | --- |
| **Number of patients with Skin Toxicity*, n (%)** | 1  (25.0) | | 2  (40.0) | 2  (33.3) | 5  (33.3) |
| **Maximum toxicity grade, n (%)** | | | | | |
| Grade 1 | 1  (25.0) | | 1  (20.0) | 2  (33.3) | 4  (26.7) |
| Grade 2 | 0 | | 1  (20.0) | 0 | 1  (6.7) |
| Grade 3 | 0 | | 0 | 0 | 0 |
| Grade 4 | 0 | | 0 | 0 | 0 |
| Grade 5 | 0 | | 0 | 0 | 0 |
| **No. of patients with Skin Toxicity* leading to study drug discontinuation** | 0 | | 0 | 0 | 0 |
| **No. of patients with Skin Toxicity* leading to study dose modification^a^** | | | | | |
| Delayed | 0 | | 0 | 0 | 0 |
| Skipped | 0 | | 0 | 0 | 0 |
| Reduced | 0 | | 0 | 0 | 0 |
| **Occurrence of Skin Toxicity*^a, b^** | | | | | |
| Step-up Dose 1 | 0 | | 0 | 0 | 0 |
| Step-up Dose 2 | 0 | | 0 | 0 | 0 |
| Step-up Dose 3 | - | | - | 0 | 0 |
| Repeat Step-up | 0 | | 0 | 0 | 0 |
| Cycle 1 Day 1 | 0 | | 0 | 2  (33.3) | 2  (13.3) |
| Cycle 1 Day 8 | 1  (25.0) | | 1  (20.0) | 0 | 2  (13.3) |
| Subsequent Doses | 0 | | 1  (20.0) | 1  (16.7) | 2  (13.3) |
| **Day of Skin Toxicity* onset relative to initial step-up dose; Median (range)** | 20  (20–20) | | 200  (19–243) | 20  (15–105) | 20  (15–243) |
| **Duration of Skin Toxicity*, days^c^ Median (range)** | 193.0  (193–193) | | 38.5  (24–53) | 23.0  (23–23) | 38.5  (23–193) |
| **Patients with Concomitant medications for Skin Toxicity*^a,d^_,_**  **n (%)** | 0 | | 2  (40.0) | 2  (33.3) | 4  (26.7) |
| **Topical** | 0 | | 2  (40.0) | 2  (33.3) | 4  (26.7) |
| Dexamethasone dipropionate | 0 | | 1  (20.0) | 0 | 1  (6.7) |
| Difluprednate | 0 | | 1  (20.0) | 0 | 1  (6.7) |
| Heparinoid | 0 | | 0 | 1  (16.7) | 1  (6.7) |
| Mometasone furoate | 0 | | 1  (20.0) | 0 | 1  (6.7) |
| Mucopolysaccharide polysulfuric acid ester | 0 | | 0 | 1  (16.7) | 1  (6.7) |
| Urea | 0 | | 1  (20.0) | 0 | 1  (6.7) |
| **Oral** |  | |  |  |  |
| Bepotastine besilate |  | | 1  (20.0) |  | 1  (6.7) |
| Emedastine fumarate |  | | 1  (20.0) |  | 1  (6.7) |
| Olopatadine hydrochloride |  | | 1  (20.0) |  | 1  (6.7) |
| **Outcome of Skin Toxicity*, n (%)** | | | | | |
| Recovered or resolved | 1  (100.0) | | 2  (66.7) | 1  (33.3) | 4  (57.1) |
| Not recovered or not resolved | 0 | | 1  (33.3) | 2  (66.7) | 3  (42.9) |
| Recovered or resolved with sequelae | 0 | | 0 | 0 | 0 |
| Recovering or resolving | 0 | | 0 | 0 | 0 |
| Fatal | 0 | | 0 | 0 | 0 |
| Unknown | 0 | | 0 | 0 | 0 |
| Missing | 0 | | 0 | 0 | 0 |

*Including Skin exfoliation, Dryskin, Pruritus, Palmar-plantar erythrodysaesthesia syndrome.

Treatment schedule: Cohort 1: 135 μg/kg SC QW, Cohort 2: 400 μg/kg SC QW, and Cohort 3: 800 μg/kg SC Q2W. ^a^Patients may appear in more than one category. ^b^Occurrence is based on the last treatment visit on or prior to the day in which the TEAE occurred. ^c^Include AEs with both start and end dates are available. ^d^All concomitant medications for Skin Toxicity* are included. Percentages calculated with the number of patients in all treated analysis set as denominator, except for the outcome of Skin Toxicity*.

QW, every week; Q2W, every 2 weeks; TEAE, treatment-emergent adverse event.

**Supplementary Table S6**: Summary of treatment-emergent adverse events of clinical interest of rash (All treated analysis set)

|  | **Cohort 1 (n=4)** | **Cohort 2**  **(n=5)** | **Cohort 3**  **(n=6)** | **Total (N=15)** |
| --- | --- | --- | --- | --- |
| **Number of patients with Rash*, n (%)** | 0 | 1 (20.0) | 2 (33.3) | 3 (20.0) |
| **Maximum toxicity grade** | | | | |
| Grade 1 | 0 | 1 (20.0) | 0 | 1 (6.7) |
| Grade 2 | 0 | 0 | 2 (33.3) | 2 (13.3) |
| Grade 3 | 0 | 0 | 0 | 0 |
| Grade 4 | 0 | 0 | 0 | 0 |
| Grade 5 | 0 | 0 | 0 | 0 |
| **No. of patients with Rash* leading to study drug discontinuation** | 0 | 0 | 0 | 0 |
| **Number of patients with Rash* leading to dose modification^a^, n (%)** | | | |  |
| Delayed | 0 | 0 | 0 | 0 |
| Skipped | 0 | 0 | 0 | 0 |
| Reduced | 0 | 0 | 0 | 0 |
| **Occurrence of Rash*^a,b^, n (%)** | | | | |
| Step-up Dose 1 | 0 | 0 | 0 | 0 |
| Step-up Dose 2 | 0 | 0 | 0 | 0 |
| Step-up Dose 3 | - | - | 0 | 0 |
| Repeat Step-up | 0 | 0 | 0 | 0 |
| Cycle 1 Day 1 | 0 | 1  (20.0) | 0 | 1  (6.7) |
| Cycle 1 Day 8 | 0 | 0 | 0 | 0 |
| Subsequent Doses | 0 | 1  (20.0) | 2  (33.3) | 3  (20.0) |
| **Day of Rash* onset relative to initial step-up dose, Median (range)** | - | 22  (9–35) | 102  (71–341) | 71  (9–341) |
| **Duration of Rash*, days^c^, Median (range)** | - | 21.0  (21–21) | 46.0  (5–87) | 21.0  (5–87) |
| **Patients with Concomitant medications for Rash*^a,d^ , n (%)** | 0 | 1  (20) | 2  (33.3) | 3  (20.0) |
| **Topical** | 0 | 1  (20) | 2  (33.3) | 3  (20.0) |
| Difluprednate | 0 | 1  (20) | 2  (33.3) | 3  (20.0) |
| Betamethasone butyrate propionate | 0 | 0 | 1  (16.7) | 1(6.7) |
| Diphenhydramine | 0 | 0 | 1  (16.7) | 1  (6.7) |
| Fluocinonide | 0 | 0 | 1  (16.7) | 1  (6.7) |
| Heparinoid | 0 | 0 | 1  (16.7) | 1  (6.7) |
| **Oral** | 0 | 1  (20) | 1  (16.7) | 2  (13.3) |
| Desloratadine | 0 | 1  (20) | 0 | 1  (6.7) |
| Fexofenadine hydrochloride | 0 | 0 | 1  (16.7) | 1  (6.7) |
| **Outcome of Rash*^a^** | | | | |
| Recovered or resolved | 0 | 1  (50.0) | 2  (66.7) | 3  (60.0) |
| Not recovered or not resolved | 0 | 1  (50.0) | 1  (33.3) | 2  (40.0) |
| Recovered or resolved with sequelae | 0 | 0 | 0 | 0 |
| Recovering or resolving | 0 | 0 | 0 | 0 |
| Fatal | 0 | 0 | 0 | 0 |
| Unknown | 0 | 0 | 0 | 0 |
| Missing | 0 | 0 | 0 | 0 |

Treatment schedule: Cohort 1: 135 μg/kg SC QW, Cohort 2: 400 μg/kg SC QW, and Cohort 3: 800 μg/kg SC Q2W. *including rash, rash maculo-papular, rash erythematous, erythema. ^a^Patients may appear in more than one category. ^b^Occurrence is based on the last treatment visit on or prior to the day in which the TEAE occurred. ^c^Includes AEs with both start and end dates are available. ^d^All concomitant medications for Rash* are included. Percentages calculated with the number of patients in all treatment analysis set as denominator, except for the outcome of Rash*.

NOTE: For Nail disorders only one patient (1 [16.7%]) was given the concomitant medication (HEPARINOID) in Cohort 3.

QW, every week; Q2W, every 2 weeks; TEAE, treatment-emergent adverse event.

**Supplementary Table S7:** Patient prior CAR-T cell therapies and response status to talquetamab

| S. No. | Treatment Group | Procedure | Best Response | Days from CAR-T to the first dose of talquetamab |
| --- | --- | --- | --- | --- |
| 1 | Cohort 1 | CAR-T(BCMA) | Stable Disease | -576 |
| 2 | Cohort 1 | CAR-T(BCMA) | Stable Disease | -154 |
| 3 | Cohort 2 | CAR-T(BCMA) | Complete Response | -601 |

Treatments: Cohort 1: 135 µg/kg QW; Cohort 2: 400 µg/kg QW; Cohort 3: 800 µg/kg Q2W.

BCMA, B-cell maturation antigen; CAR-T, Chimeric Antigen Receptor T-cell therapy; QW, every week; Q2W, every 2 weeks.
